# Is mental health co-morbidity an influencing factor in the health service utilisation of women with diabetes mellitus?

**DOI:** 10.1371/journal.pone.0272041

**Published:** 2022-08-08

**Authors:** Tracey Oorschot, Jon Adams, David Sibbritt

**Affiliations:** School of Public Health, University of Technology Sydney, Sydney, New South Wales, Australia; PLOS: Public Library of Science, UNITED KINGDOM

## Abstract

Diabetes Mellitus, affecting nearly half a billion people worldwide, is a substantial global public health issue. Although diabetes predominantly affects men, women with diabetes have specific risks and self-management characteristics. Women have a higher risk of either presenting with or developing depression or anxiety, as well as being high users of complementary medicine which can create clinical governance issues. In spite of these known gender differences, limited research has explored gender-specific diabetes care, especially health service use patterns. As increasing attention has turned to supporting people with diabetes to successfully self-manage their diabetes, it is important that we understand how women with diabetes are using health services, and if their specific risk profile is influencing their health care choices. Our study sought to examine the relationship between mental health status and the patterns of conventional and complementary medicine health service use by women diagnosed with diabetes mellitus. Our results showed that women with diabetes and *any* mental health co-morbidity were more likely to visit their general practitioner more frequently or use herbal medicine than those without a mental health co-morbidity. Women with depression and anxiety were also less likely to consult a physiotherapist and those with anxiety less likely to consult a podiatrist over time when compared to the other mental health groups.

## Introduction

Diabetes Mellitus can have significant impacts upon health outcomes, including increased risk of physical and/or mental health co-morbidities, in turn potentially reducing both quality of life and life expectancy [1, 2]. Providing and supporting optimal diabetes management continues to be a key driver of diabetes care [3, 4]. Supporting primary care is also another key component of diabetes care, given that the majority of diabetes patients work predominantly with their primary care physician for ongoing diabetes management [4–6].

Previous research has identified a number of health care access and servicing issues within primary diabetes care [4, 7, 8]. Of prime concern is supporting and improving an individual’s capacity to successfully self-manage their diabetes, to prevent or ameliorate diabetes complications and improve/maintain quality of life across the life span [3, 9, 10]. Historically, primary attention was given to reducing risk factors concerning serious physical health co-morbidities [6] such as cardiovascular disease, neuropathy and nephropathy. As diabetes can be a lifelong disease, it became apparent that the impacts of living with diabetes can also substantially affect mental health [6, 9–11]. Mental health co-morbidity has also been shown to influence health behaviours [8, 11], resulting in an increase in this field of research. People with diabetes and either anxiety or depression have been shown to have more difficulty in maintaining self-management routines, such as exercise, diet, medication and blood glucose monitoring, placing this cohort at increased risk of poor glycemic control and lowered health outcomes [12, 13]. Additionally, many people with diabetes do not receive standard levels of care, such as completing annual cycles of review with a primary care physician, eye health exams with an optometrist and so forth [14, 15]. Although research to determine factors influencing suboptimal diabetes care has been undertaken, this has mostly concentrated on sociodemographic issues (e.g. geographic location, individual socioeconomic status, service availability and cost). The role of a mental health co-morbidity upon health service utilisation relevant to diabetes care has been largely unexplored.

Mental health research has established that people with any mental health condition often do not seek help or receive adequate mental health care from health care professionals [16]. This finding, together with it being known that a mental health co-morbidity can lead to poorer diabetes health outcomes, prompted health care professionals to place increased importance on screening for and monitoring the most commonly experienced forms of mental health conditions for people with diabetes–anxiety or depression [6]. Whilst diabetes research has replicated general mental health findings in that people with diabetes also often do not receive adequate mental health care [6], it is not known if or how anxiety or depression influences health service utilisation related to diabetes care. As standard diabetes care typically requires consulting with a variety of health care professionals (e.g. pharmacist, podiatrist, dietitian, physiotherapist, exercise physiologist, etc), it is important to discover if a mental health co-morbidity is another factor influencing health service engagement, and in turn, diabetes self-management [17]. Furthermore, as with women’s medical research in general [18], limited research has explored gender-specific self-management risks within the diabetes population. despite there being known gender differences.

Early studies have shown women to be almost twice as likely to have co-morbid anxiety, and almost 2.5 times more likely to have co-morbid depression compared to men [11], increasing the importance of understanding the role of mental health co-morbidity upon self-management behaviours for women with diabetes. Another difference of significance, is that women with either diabetes or depression or anxiety are high users of complementary medicine (CM) [19, 20]. CM use can also be accompanied with low to high risk factors of harm due to self-prescription, such as economic losses stemming from using ineffective CM products to negative health consequences arising from contraindications [21]. Additionally, non-disclosure of CM use to health care professionals is also common, which decreases clinical governance opportunities to reduce risks [19, 22]. Clinical governance can be defined in a number of ways. For the purpose of this article, clinical governance refers to risk management at the clinical level, with the overall intent of providing safe and quality care to mitigate adverse health outcomes [17]. Being aware of and understanding gender differences becomes an important factor in diabetes care, both in terms of targeted clinical governance to manage risks, as well as for developing clinical approaches that foster a therapeutic alliance of relevant providers.

Furthermore, diabetes research principally focuses upon a single mental health status within the same diabetes population, especially depression, despite known associations between anxiety and metabolic diseases [11]. Diabetes research to date has also failed to adequately explore if or how depression or anxiety might influence health service use. To address these research gaps, this study focuses on the health characteristics and health service use (conventional or CM health service practitioner consultation or practice/product use) of 4 groups of women: those with diabetes; those with diabetes and co-morbid depression; those with diabetes and anxiety; and those with diabetes *and both* depression and anxiety.

## Materials and methods

### Research design and ethics

This paper reports the results of a secondary analysis drawing from the Australian Longitudinal Study on Women’s Health (ALSWH). ALSWH commenced in 1996 surveying three age cohorts–*Young* (18–23 years), *Mid* (45–50 years) and *Old* (70–75 years) approximately every three years. ALSWH participant recruitment involved randomly sampling individuals from within each of the age groups from the Medicare Benefits Schedule database, with women living in rural and remote areas oversampled to achieve a nationally representative sample [23]. Ethics approval for ALSWH was obtained by the Human Research Ethics Committees at the University of Newcastle and the University of Queensland. A formal Expression of Interest (EOI A842) was presented to the ALSWH committee with permission granted to conduct the secondary analyses reported in this study. Data obtained was de-identified and restricted to the cohort and survey data discussed in this paper.

### Study population

This secondary analysis focuses on the ALSWH mid cohort (women born between 1946–51). Eight surveys have been reported for this cohort to date, with participants aged between 45–50 years old at survey 1 in 1996 through to being aged 68–73 at survey 8 in 2019. This study reports results from two separate statistical analyses drawing from surveys 5, 6 and 7. Surveys 1–4 and survey 8 were excluded from the study due to the omission of questions regarding CM use, that were contained in surveys 5–7. Participants were aged 56–61 years old in survey 5 (2007), 59–64 years old in survey 6 (2010), and 62–67 years old in survey 7 (2013). Survey 7 (n = 11,290) was utilised to conduct cross sectional analyses, of which 8,694 participants completed the question concerning diabetes mellitus status. Surveys 5, 6 and 7 (n = 10,509, n = 9,902, n = 9,851, respectively) were utilised to conduct the longitudinal analysis.

### Study sample

Drawing from the initial ALSWH survey samples described above, women who self-reported a diabetes diagnosis (either Type 1 or Type 2 diabetes mellitus) were selected for further analysis. This equated to 693 (7.03%) participants in survey 5, 781 (7.89%) in survey 6, and 804 (9.25%) in survey 7. Tables 2–4 reports the final sample sizes of participants who completed the survey questions pertinent to mental health status and health service use that are the subject of this paper.

### MH status

To determine clinical levels of depression or anxiety we employed the Center for Epidemiologic Studies Depression scale [CESD-10: 24] and the Goldberg Anxiety & Depression Scale [GADS: 25] respectively as utilised within the ALSWH data sets from surveys 5 through 7. CESD-10 scores range from 0–30, with higher scores indicating higher levels of depression. Using the conventional threshold of 10 to identify clinical levels of depression, women who scored 10 or more were classified as depressed (yes), with those scoring less than 10 classified as not depressed (no). GADS Scores range from 0 to 21, with scores of 5 or more indicating clinical levels of anxiety. Women who scored 5 or more were classified as having anxiety (yes), whilst those scoring less than 5 were classified as not having anxiety (no). Women who met the criteria for both clinical depression and clinical anxiety were classified as having both depression and anxiety (yes or no).

### Health service use

The ALSWH survey asked women about their conventional and complementary medicine consultation patterns or practice/product use in the previous 12 months. Questions focused upon conventional health service use included the number of visits to medical practitioners such as a general practitioner (GP)/primary care physician, specialist doctor (secondary health service), hospital doctor (tertiary health service), and an allied health practitioner such as a mental health worker (defined as either a counsellor, psychologist or social worker), physiotherapist, among others and product use concerning prescription medicines. Similarly, questions focused upon CM health service use included practitioner consultations with a naturopath/herbalist, osteopath, chiropractor, etc., and practice/product use such as vitamins/minerals, herbal medicines or yoga. The results from GP visits were further grouped into sub-categories–low (<5 visits annually), medium (5–12 visits annually) and high (13+ visits annually).

### Statistical analyses

All statistical analyses were conducted using the statistical software package Stata, version 16.1 [26]. Initial bivariate analyses using chi-square tests were conducted to determine the associations between mental health status and a diabetes diagnosis. The outcome variables pertained to conventional or CM health service use. Frequency of conventional or CM practitioner or practice/product use was estimated using two-way tables. Chi-squared tests were conducted to determine the significance of mental health status and associated health service use among those with diabetes. A generalized estimating equation (GEE) model was used to examine the longitudinal association between mental health status and consultation with conventional or CM health care practitioners, or conventional or CM practice/product use. The GEE method was developed to produce population-averaged regression model estimates when analysing repeated measures with non-normal response variables, focusing on average changes in response over time and the impact of covariates on these changes. So, for this study, the GEE model allows for the analysis of the data collected longitudinally, thus reflecting the relationship between the longitudinal development of mental health status and the longitudinal development of the health service use variables over time. A *p*-value < .05 was considered statistically significant.

### Covariates

A range of socio-demographic, health behaviour and health status variables that are known to be contributing factors to diabetes or mental health status were examined as potential cofounders (see Table 2). The socio-demographic variables considered included area of residence; marital, retirement or employment status; and whether the participant held private health insurance (hospital or general treatment coverage) or had concessional health service eligibility (health care card). Health behaviours considered included level of alcohol use, exercise and smoking. Health statuses considered included state of general health, Body Mass Index (BMI), stress level related to own health, and if the participant had heart disease or hypertension.

These variables were considered against each mental health category, with the longitudinal analyses adjusted accordingly. Covariates included for co-morbid depression included marital status, private health insurance (hospital), exercise level, stress level, and general health. For women with anxiety, health care card status, general health and stress level. Finally, for women with both depression and anxiety, covariates included area of residence, marital status, private health insurance (hospital), general health and BMI.

## Results and discussion

### Cross-sectional results

Of the women who reported a diabetes diagnosis (n = 789, n = 799, n = 753 respectively), 233 (29.5%) were determined to have clinical levels of depression, 345 (43.2%) were determined to have clinical levels of anxiety, and 200 (26.6%) were determined to have both depression and anxiety. A diabetes diagnosis was found to be significantly associated (*p* <0.001) with clinical depression or anxiety or depression and anxiety (see Table 1).

**Table 1:** Associations between mental health status and diabetes mellitus for 8694 participants in survey 7 (2013).

Mental Health Status	Diabetes Status		
	No (n = 7890)	Yes (n = 804)	p-value
***Depression***			<0.001
No	6575 (83.33)	556 (69.15)	
Yes	1235 (15.66)	233 (28.98)	
Missing	80 (1.01)	15 (1.87)	
***Anxiety***			<0.001
No	5387 (68.28)	454 (56.47)	
Yes	2464 (31.22)	345 (42.91)	
Missing	39 (0.50)	5 (0.62)	
**Anxiety & Depression**			<0.001
No	6545 (82.95)	553 (68.78)	
Yes	1013 (12.84)	200 (24.88)	
Missing	332 (4.21)	51 (6.34)	

### Associations between mental health status and socio-demographic, health behaviour and health status

As Table 2 shows, socio-demographic variables of significance (*p*<0.05) for all mental health groups (depression, anxiety or depression and anxiety) included marital, retirement, and employment status, and private hospital insurance (hospital or ancillary coverage). Similarly, health behaviours and health status factors of significance (*p*<0.05) included level of exercise, general health status, body mass index (BMI) category, and stress level concerning own health. Health card status and the presence of hypertension were significantly (*p*<0.05) associated for women with anxiety, or depression and anxiety only. Further analysis of these variables of significance, showed associative differences when comparing women with diabetes to those with co-morbid mental health, as well as within the mental health status groups (depression, anxiety or depression and anxiety).

**Table 2:** Socio-demographic, health behaviour and health status characteristics of women with diabetes mellitus by mental health status.

Characteristic	Mental Health Status								
	Depression (n = 789) ^1^			Anxiety (n = 799) ^2^			Depression & Anxiety (n = 753) ^3^		
	No (n = 556)	Yes (n = 233)	p-value	No (n = 454)	Yes (n = 345)	p-value	No (n = 553)	Yes (n = 200)	p-value
	n (%)	n (%)		n (%)	n (%)		n (%)	n (%)	
**Socio-demographic**									
***Area of residence***			*0*.*116*			*0*.*581*			*0*.*093*
Major cities	208 (37.7)	70 (30.0)		164 (36.4)	116 (33.6)		207 (37.7)	59 (29.5)	
Inner regional	225 (40.8)	104 (44.7)		190 (42.2)	146 (42.3)		224 (40.8)	88 (44.0)	
Outer reg/remote/ v remote/os	119 (21.5)	59 (25.3)		96 (21.3)	83 (24.1)		118 (21.5)	53 (26.5)	
***Marital Status***			*<0*.*001*			*0*.*023*			*0*.*001*
Married/de facto	415 (75.0)	143 (61.6)		335 (74.6)	227 (66.0)		412 (74.9)	123 (61.5)	
Separated/divorced/widowed	121 (21.9)	82 (35.3)		100 (22.3)	106 (30.8)		121 (22.0)	70 (35.0)	
Never married	17 (3.1)	7 (3.0)		14 (3.1)	11 (3.2)		17 (3.1)	7 (3.5)	
***Retirement status***			*0*.*001*			*0*.*002*			*0*.*001*
Not retired	116 (21.3)	28 (12.8)		98 (22.1)	48 (14.7)		116 (21.4)	21 (11.3)	
Partially retired	64 (11.7)	15 (6.9)		53 (11.9)	25 (7.7)		63 (11.6)	14 (7.5)	
Retired	366 (67.0)	175 (80.3)		293 (66.0)	253 (77.6)		364 (67.0)	151 (81.2)	
***Employed***			*<0*.*001*			*<0*.*001*			*<0*.*001*
No	220 (39.7)	61 (26.5)		186 (41.4)	98 (28.7)		220 (39.9)	50 (25.3)	
Yes	334 (60.3)	169 (73.5)		263 (58.6)	243 (71.3)		331 (60.1)	148 (74.7)	
***Health care card status*** ^a^			*0*.*078*			*0*.*001*			*0*.*018*
No	216 (39.1)	75 (32.5)		190 (42 2)	104 (30.4)		216 (39.3)	59 (29.8)	
Yes	336 (60.9)	156 (67.5)		260 (57.8)	238 (69.6)		334 (60.7)	139 (70.2)	
***PHI status (hospital)*** ^b^			*<0*.*001*			*0*.*013*			*<0*.*001*
No	230 (41.6)	130 (56.3)		190 (42.1)	175 (51.0)		229 (41.6)	112 (56.3)	
Yes	323 (58.4)	101 (43.7)		261 (57.9)	168 (49.0)		322 (58.4)	87 (43.7)	
***PHI status (ancillary)*** ^c^			*0*.*001*			*0*.*040*			*0*.*001*
No	253 (45.8)	299 (54.2)		210 (46.6)	185 (53.9)		252 (45.8)	119 (59.8)	
Yes	137 (59.0)	95 (41.0)		241 (53.4)	158 (46.1)		298 (54.2)	80 (40.2)	
**Health Behaviour**									
***Alcohol use***			*0*.*057*			*0*.*098*			*0*.*179*
Low risk drinker	195 (35.9)	64 (28.5)		163 (36.7)	98 (29.5)		195 (36.0)	56 (29.0)	
Non-drinker/rarely drinks	333 (61.2)	149 (66.2)		267 (60.1)	220 (66.3)		330 (61.0)	129 (66.8)	
Risky/high risk drink	16 (2.9)	12 (5.3)		14 (3.2)	14 (4.2)		16 (2.96)	8 (4.2)	
***Exercise level***			*<0*.*001*						*<0*.*001*
Inactive	124 (22.9)	94 (42.2)		99 (22.9)	121 (36.0)	*0*.*001*	123 (22.9)	79 (40.9)	
Low	144 (26.6)	51 (22.9)		115 (26.6)	82 (24.4)		144 (26.7)	45 (23.3)	
Moderate	107 (19.8)	32 (14.3)		86 (19.9)	53 (15.8)		105 (19.5)	30 (15.6)	
High	166 (30.7)	46 (20.6)		132 (30.6)	80 (23.8)		166 (30.8)	39 (20.2)	
***Smoking***			*0*.*950*			*0*.*831*			*0*.*754*
No	514 (92.9)	215 (93.1)		421 (93.4)	317 (92.9)		512 (93.1)	183 (92.4)	
Yes	39 (7.1)	16 (6.9)		30 (6.6)	24 (7.1)		38 (6.9)	15 (7.6)	
**Health status**									
***General health***			*<0*.*001*			*<0*.*001*			*<0*.*001*
Poor/Fair/Good	420 (75.5)	215 (93.5)		327 (72.0)	316 (92.4)		417 (75.4)	190 (96.4)	
Very good/excellent	136 (24.5)	15 (6.5)		127 (28.0)	26 (7.6)		136 (24.6)	7 (3.6)	
***BMI Category***			*0*.*003*			*0*.*001*			*<0*.*001*
Underweight/healthy weight	76 (14.5)	17 (7.7)		63 (14.6)	32 (9.9)		76 (14.5)	12 (6.3)	
Overweight	148 (28.1)	50 (22.6)		129 (29.9)	69 (21.4)		146 (27.9)	40 (21.1)	
Obese	302 (57.4)	154 (69.7)		239 (55.5)	222 (68.7)		301 (57.5)	138 (72.6)	
***Heart disease***			*0*.*604*			*0*.*430*			0.615
No	474 (85.6)	196 (84.1)		389 (86.1)	290 (84.1)		471 (85.5)	168 (84.0)	
Yes	80 (14.4)	37 (15.9)		63 (13.9)	55 (15.9)		80 (14.5)	32 (16.0)	
***Hypertension***			*0*.*320*			*0*.*005*			*0*.*066*
No	211 (38.1)	80 (34.3)		189 (41.8)	111 (32.2)		211 (38.3)	62 (31.0)	
Yes	343 (61.9)	153 (65.7)		263 (58.2)	234 (67.8)		340 (61.7)	138 (69.0)	
***Stressed about own health***			*<0*.*001*			*<0*.*001*			*<0*.*001*
Not stressed	218 (39.2)	20 (8.7)		196 (43.6)	41 (11.9)		216 (39.1)	15 (7.6)	
Somewhat/ moderately stressed	323 (58.1)	156 (67.5)		242 (53.8)	244 (71.2)		322 (58.2)	132 (66.3)	
Very/extremely stressed	15 (2.7)	55 (23.8)		12 (2.6)	58 (16.9)		15 (2.7))	52 (26.1)	

Notes:

^a^: health care card = eligibility for concessional health services within Australia

^b^: PHI hospital = private health insurance coverage for treatment services in Australian hospitals by a private health care provider

^c^: PHI ancillary = private health insurance coverage for general treatment (e.g. physiotherapy) by a private health care provider

^1^: there were 15 non-responses to the question on depression

^2^: there were 5 non-responses to the question on anxiety

^3^: there were 51 non-responses to the questions on depression and anxiety

Of those with co-morbid mental health, women with depression and anxiety had the highest reported rates of retirement, employment or possessing a health care card. Conversely, women with diabetes more commonly reported having private health insurance than those with co-morbid mental health. Similarly considering health behaviours and health status characteristics, more women who reported exercising to some degree had diabetes, compared to those with co-morbid mental health, with women with depression mostly reporting not exercising. On the other hand, those with co-morbid mental health reported higher rates of obesity or hypertension, compared to those with diabetes. Most women reported a somewhat to moderate stress level or a poor to good general health status but was higher for women with co-morbid mental health than those with diabetes. The highest stress or lowest general health statuses were both associated with depression and anxiety.

### Prevalence of health service use

From Table 3, the practitioner group consultations significantly associated (*p*<0.05) across all the MH groups (diabetes, depression, anxiety or depression and anxiety) included GP, hospital doctor, mental health worker and dietitian. Most women with co-morbid mental health consulted with a GP 5–12 times a year, whereas those with diabetes mostly consulted a GP less than 5 times annually. Women with co-morbid mental health also more commonly reported visiting tertiary or allied health care than those with diabetes.

**Table 3:** Prevalence of health service use of women with diabetes mellitus by mental health status.

Health Service Use (yes)		MH Status								
		Depression (n = 789)^1^			Anxiety (n = 799) ^2^			Depression & Anxiety (n = 753) ^3^		
		No (n = 556)	Yes (n = 233)	p-value	No (n = 454)	Yes (n = 345)	p-value	No (n = 553)	Yes (n = 200)	p-value
		n (%)	n (%)		n (%)	n (%)		n (%)	n (%)	
**Practitioners**										
*Conventional*										
***GP***:	Low	275 (49.5)	63 (27.0)	*<0*.*001*	238 (52.5)	102 (29.6)	*<0*.*001*	274 (49.5)	50 (25.0)	*<0*.*001*
	Med	234 (42.1)	115 (49.4)		177 (39.1)	178 (51.6)		232 (41.9)	103 (51.5)	
	High	47 (8.4)	55 (23.6)		38 (8.4)	65 (18.8)		47 (8.5)	47 (23.5)	
***Hospital doctor***		156 (28.1)	102 (43.8)	*<0*.*001*	111 (24.6)	149 (43.2)	*<0*.*001*	154 (27.9)	90 (45.0)	*<0*.*001*
***Specialist doctor***		342 (61.6)	161 (69.1)	*0*.*046*	262 (58.0)	245 (71.0)	*<0*.*001*	340 (61.5)	137 (68.5)	*0*.*082*
***Physiotherapist***		137 (24.9)	66 (28.1)	*0*.*309*	102 (22.8)	102 (29.8)	*0*.*025*	135 (24.7)	56 (28.1)	*0*.*345*
***Mental health worker***		25 (4.5)	45 (19.6)	*<0*.*001*	18 (4.0)	55 (16.2)	*<0*.*001*	25 (4.6)	41 (20.9)	*<0*.*001*
***Community Nurse***		173 (31.3)	90 (39.3)	*0*.*032*	123 (27.4)	143 (41.9)	*<0*.*001*	171 (31.1)	84 (42.6)	*0*.*004*
***Optician***		437 (79.0)	165 (71.4)	*0*.*022*	350 (77.8)	257 (75.1)	*0*.*386*	434 (78.9)	143 (72.2)	*0*.*055*
***Dietitian***		148 (26.9)	89 (38.9)	*0*.*001*	116 (26.0)	124 (36.4)	*0*.*002*	147 (26.8)	80 (40.4)	*<0*.*001*
***Podiatrist***		279 (50.4)	123 (53.2)	*0*.*461*	227 (50.4)	177 (51.6)	*0*.*746*	276 (50.1)	106 (53.3)	*0*.*442*
***Dentist***		330 (59.8)	112 (48.3)	*0*.*003*	264 (58.7)	184 (53.6)	*0*.*158*	329 (59.9)	96 (48.0)	*0*.*004*
*Complementary Medicine*										
***Massage Therapist***		110 (19.9)	39 (16.8)	*0*.*310*	83 (18.5)	66 (19.2)	*0*.*814*	109 (19.8)	36 (18.0)	*0*.*570*
***Naturopath/herbalist***		30 (5.5)	19 (8.2)	*0*.*147*	22 (4.9)	27 (7.9)	*0*.*088*	30 (5.5)	17 (8.5)	*0*.*130*
***Chiropractor***		73 (13.2)	30 (12.9)	*0*.*888*	61 (13.6)	43 (12.5)	*0*.*665*	73 (13.3)	22 (11.0)	*0*.*399*
***Osteopath***		20 (3.6)	7 (3.1)	*0*.*688*	13 (2.9)	14 (4.1)	*0*.*358*	20 (3.6)	6 (3.1)	*0*.*691*
***Acupuncturist***		30 (5.4)	18 (7.8)	*0*.*212*	23 (5.1)	25 (7.3)	*0*.*211*	30 (5.5)	17 (8.5)	*0*.*127*
**Practice or Products**										
*Conventional*										
***Prescription Medicine***		531 (95.7)	225 (97.4)	*0*.*250*	430 (95.6)	333 (97.1)	*0*.*264*	528 (95.6)	194 (98.0)	*0*.*138*
*Complementary Medicine*										
***Vitamins/minerals***		430 (77.8)	190 (81.9)	*0*.*194*	344 (76.3)	281 (81.9)	*0*.*054*	428 (77.8)	164 (82.4)	*0*.*172*
***Yoga/meditation***		96 (17.3)	47 (20.2)	*0*.*331*	73 (16.1)	73 (21.3)	*0*.*064*	96 (17.4)	39 (19.6)	*0*.*494*
***Herbal medicines***		144 (26.1)	79 (34.0)	*0*.*024*	112 (24.9)	113 (32.8)	*0*.*014*	143 (26.1)	69 (34.7)	*0*.*021*
***Aromatherapy oils***		100 (18.1)	47 (20.3)	*0*.*483*	70 (15.6)	79 (22.9)	*0*.*009*	100 (18.21)	44 (22.0)	*0*.*245*
***Chinese medicines***		41 (7.4)	13 (5.6)	*0*.*359*	27 (6.0)	27 (7.8)	*0*.*302*	41 (7.4)	12 (6.0)	*0*.*499*

Notes:

^1^: there were 15 non-responses to the question on depression

^2^: there were 5 non-responses to the question on anxiety

^3^: there were 51 non-responses to the questions on depression and anxiety

Consulting with a specialist doctor was significantly associated (*p*<0.05) with depression or anxiety, a physiotherapist with anxiety, an optician with depression, and a dentist with depression or depression and anxiety. Women with anxiety reported a higher proportion of consultation with a specialist doctor and physiotherapist. Conversely, visiting an optician showed a higher proportion of women reporting diabetes. Likewise, more women with diabetes saw a dentist. Consulting with a podiatrist was the only practitioner group to have no significant associations (*p*<0.05).

There were no significant associations (*p*<0.05) for consultation with any CM practitioner group. For practice/product use, herbal medicine use was significantly associated (*p*<0.05) across all mental health groups, with women with co-morbid mental health having higher rates of herbal medicine use, than those with diabetes. Aromatherapy use was significantly associated (*p*<0.05) with anxiety, whereas women with depression and anxiety showed a slightly higher proportion of herbal medicine use.

### Longitudinal results

#### Associations between mental health status and health service use

As Table 4 shows, the practitioner groups that were significantly associated (*p*<0.05) with all mental health groups, related to consultation with a mental health worker or a GP. Similarly, for practice/product use, herbal medicine use was also significantly associated (*p*<0.05) with all mental health groups. Consulting with a specialist doctor was significantly associated (*p*<0.05) with depression. Visiting a podiatrist or using aromatherapy was significantly associated (*p*<0.05) with anxiety, whereas consulting with an optician or using prescription medicine was significantly associated (*p*<0.05) with both depression and anxiety. Finally, those who consulted with a naturopath/herbalist was significantly associated (*p*<0.05) with anxiety or both depression and anxiety.

**Table 4:** The longitudinal association between mental health status and health service use by women with DM mellitus (2007–2013), as determined by generalized estimating equations (GEEs).

Health Service Use (yes)		MH Status (yes)								
		Depression ^a^			Anxiety ^b^			Depression & Anxiety ^c^		
		OR	95% CI	p-value	OR	95% CI	p-value	OR	95% CI	p-value
**Practitioners**										
*Conventional Practitioners*										
***GP***: (low)	Med	1.27	.987, 1.636	*0*.*063*	1.37	1.116, 1.686	*0*.*003*	1.50	1.169, 1.897	*0*.*001*
	High	1.58	1.098, 2.284	*0*.*014*	1.70	1.234, 2.352	*0*.*001*	2.06	1.473, 2.879	*<0*.*001*
***Specialist doctor***		0.77	.610, .984	*0*.*036*	x			x		
***Mental health worker***		2.21	1.586, 3.092	*<0*.*001*	2.10	1.496, 2.949	*<0*.*001*	2.88	2.101, 3.957	*<0*.*001*
***Optician***		x				x		0.75	.593, 0.944	*0*.*015*
***Podiatrist***		x			0.76	.627, .924	*0*.*006*	x		
*Complementary Practitioners*										
***Naturopath/herbalist***		x			1.47	1.006, 2.135	*0*.*047*	1.76	1.177, 2.581	*0*.*006*
**Practice or Products**										
*Conventional Medicine*										
***Prescription Medicine***		x				x		1.76	1.049, 2.956	*0*.*032*
*Complementary Medicine*										
***Herbal medicines***		1.53	1.210, 1.931	*<0*.*001*	1.38	1.112, 1.724	*0*.*004*	1.53	1.192, 1.953	*<0*.*001*
***Aromatherapy oils***		x			1.40	1.098, 1.790	*0*.*007*			

Note:

^a^: adjusted for—marital status, private health insurance (hospital), exercise level, general health, stress level (regarding health)

^b^: adjusted for—health care card status, general health, stress level (regarding health)

^c^: adjusted for—area of residence, marital status, private health insurance (hospital), general health, BMI category

Women with depression were most likely to consult with a mental health worker, but less likely to see a specialist doctor (*OR* = 0.77, [95% CI: 0.61, 0.98]). Similarly, women with anxiety were also most likely to consult with a mental health worker, as well as using aromatherapy oils, but less likely to see a podiatrist (*OR* = 0.76, [95% CI: 0.63, 0.92]). Lastly, women with depression and anxiety had the highest likelihood of consulting with a mental health worker. Women with depression and anxiety were also less likely to consult with an optician (*OR* = 0.75, [95% CI: 0.59, 0.94]), but more than 1.5 times more likely to use prescription medicines and had the highest likelihood of consulting with their GP 7 or more times annually (twice as likely) or a naturopath (almost twice as likely).

### Discussion

The study reported here is the first to explore whether the presence of a mental health co-morbidity influences health service use for women with diabetes. The results suggest that women with a mental health co-morbidity are using different health services compared to those with diabetes only. As women with diabetes are at increased risk of developing depression or anxiety, these results go towards understanding how best to develop diabetes care for this specific cohort.

Primary care clinics are known to be essential health services for people with a mental health concern [27], especially women [28, 29]. Our study found that women with any mental health co-morbidity visited their GP more often than those with diabetes alone. Although it is known that women with chronic disease are high users of primary care [17, 30], engagement with health care professionals to address mental health concerns remains problematic [16, 31]. Therefore, this finding is an important discovery as GPs are an integral point of contact for diabetes care [5, 32] and mental health screening is not always undertaken during routine appointments [11, 33]. Furthermore, the presence of either depression or anxiety is a known risk to optimal diabetes management [11, 33] and it is critical that GPs have the capacity to screen for and case manage any mental health needs in diabetes patients [4, 34].

Allied health clinicians are another essential part of diabetes care [3, 10, 35]. Our study found significant results pertaining to physiotherapist, optician and podiatrist consultation patterns. From the cross-sectional analysis, consulting with a physiotherapist was associated with having anxiety, whilst the longitudinal analysis did not show any significant associations. However, as people with depression are just as likely to have somatic symptoms and seek physiotherapy as those with anxiety [36], it remains unclear if these results indicate that women with anxiety are more likely than those with depression to consult with a physiotherapist. Additionally, physiotherapists have called for greater treatment focus concerning mental health factors due to the known links between mental and physical health from treating large numbers of patients with chronic diseases like diabetes [36, 37]. This places increased importance on further exploring the role of mental health co-morbidity to understand the influence of co-morbid depression or anxiety upon consultation behaviours.

The cross-sectional analysis also revealed that women with depression were the only mental health group significantly associated with consulting an optician, and that consultation rates were higher for those with diabetes. People with depression or anxiety are known to have decreased help-seeking behaviours [27], with the longitudinal analysis showing significance for women with both depression and anxiety. This does not reveal whether the presence of depression or anxiety is a potential factor affecting engagement with this practitioner group. Furthermore, as people with diabetes are encouraged to seek regular eye care as part of their ongoing diabetes management [9, 10], you would expect significant associations with this practitioner group no matter the patient’s mental health status. We did not find this result, warranting further examination regarding if or how the presence of depression or anxiety influences engagement with an optician.

Regular foot care is another essential component of ongoing diabetes management [9, 10]. We did not find any significant results regarding podiatry consultation from the cross-sectional analysis but did find that women with anxiety were less likely to consult a podiatrist over time. Again, the lack of available data makes it difficult to ascertain if the presence of anxiety is the contributing factor affecting podiatry consultation. Similar to physiotherapy, people with either depression or anxiety are known to consult with podiatrists as they are at increased risk of having enhanced foot pain due to mental health co-morbidity [38]. Without further examination of the factors influencing podiatrist consultation for people with diabetes, any suggestion that the presence of anxiety decreases the likelihood of women with diabetes consulting with a podiatrist is speculative.

With regard to practice and product use, we identified that herbal medicine use was more likely for those with a mental health co-morbidity compared to those with diabetes alone. Previous research has revealed people living with diabetes [39, 40] or depression or anxiety are high users of herbal medicines [41]. Our results indicate that the presence of either depression or anxiety is influencing herbal medicine use more so than the presence of diabetes [41]. Women with anxiety were the only mental health group associated with using aromatherapy. This result is unsurprising given that aromatherapy is a known treatment choice for people with anxiety [42]. What was unexpected was the lack of a significance for vitamin/mineral use for any mental health group, as people with either depression or anxiety are known to be high users of this CM product [43]. This suggests that the presence of diabetes might be influencing vitamin/mineral use. Again, this suggestion is speculative and requires further data to examine if the presence of diabetes is influencing supplement use for women with co-morbid depression or anxiety.

Finally, considering clinical governance issues, women are known to engage in both conventional and CM health service use at the same time [41, 44]. Our findings identified women with either depression or anxiety were just as likely to use herbal medicines as they were to see a GP. The implication of this is that women with diabetes and co-morbid depression or anxiety are just as likely to be working with a CM practitioner as a conventional practitioner, elevating the need for diabetes care to take a coordinated approach across disciplines both conventional and CM. Furthermore, people who use CM do not generally disclose this use to conventional practitioners [19], as well as self-prescription of CM being common which increases risk of harm such as contra-indication [41]. As women are also high users of CM [19], it is imperative that the inclusion of CM practitioners who might be ideally positioned to provide clinical governance for this specific cohort [45–47], forms part of consideration when designing multi-disciplinary diabetes care teams.

There are limitations to this study. The ALSWH survey did not ask if health service use was directly related to diabetes care, so it cannot be generalised to the broad diabetes population. The survey also relies upon self-report which is associated with recall bias and potential inaccuracies regarding participant responses. Cross-sectional analyses can also not be drawn upon to establish causal relationships between variables, as well as secondary analyses being limited to the data generated by the primary research. The ALSWH survey was designed to cover a large variety of health and wellbeing topics, rather than specifically targeting diabetes and mental health. As such, this study focuses on the most common mental health conditions experienced by women living with diabetes–anxiety or depression and does not analyse other mental health conditions (e.g. bipolar mood disorder) or factors (e.g. personality traits).

Notwithstanding this, the research topic is innovative in so far that diabetes research has not previously examined health service use (including both conventional and CM practitioners) in conjunction with mental health status, particularly analysing multiple mental health statuses from the same diabetes population. Our study is also strengthened by drawing upon an established large nationally representative cohort study. In addition, validated psychological scales have been used to determine clinical levels of depression or anxiety rather than using the self-reported responses, which increases the likelihood of a true representation of a participant’s psychological status.

## Conclusions

The present study provides valuable insights into the role of mental health co-morbidity upon health service use. However, limited research exists to help explain some of our findings. Further quantitative research that can provide targeted analysis of health service utilisation of people living with diabetes, together with qualitative research than can explore influences upon the conventional and complementary medicine health care choices of women with diabetes is warranted. Additionally, given the propensity of CM use amongst women with diabetes or depression or anxiety, and previous CM research highlighting the non-disclosure of this use to conventional practitioners, it is crucial that we adequately understand how women with diabetes are engaging with *both* conventional and CM practitioners. Further research in this area can also explore the appropriateness of multi-disciplinary diabetes care teams (both conventional and CM practitioners), to provide clinical governance to reduce known risks with CM use, as well as support optimal diabetes self-management.
